# Forward lunge before and after anterior cruciate ligament reconstruction: Faster movement but unchanged knee joint biomechanics

**DOI:** 10.1371/journal.pone.0228071

**Published:** 2020-01-24

**Authors:** Tine Alkjær, Kenneth B. Smale, Teresa E. Flaxman, Ida F. Marker, Erik B. Simonsen, Daniel. L. Benoit, Michael R. Krogsgaard

**Affiliations:** 1 Department of Biomedical Sciences, University of Copenhagen, Copenhagen, Denmark; 2 School of Human Kinetics, University of Ottawa, Ottawa, ON, Canada; 3 School of Rehabilitation Sciences, University of Ottawa, Ottawa, ON, Canada; 4 Department of Neuroscience, University of Copenhagen, Copenhagen, Denmark; 5 Section for Sports Traumatology, M51, Bispebjerg and Frederiksberg Hospital, Copenhagen, Denmark; University of Memphis, UNITED STATES

## Abstract

The forward lunge (FL) may be a promising movement to assess functional outcome after ACL reconstruction. Thus, we aimed to investigate the FL movement pattern before and after ACL reconstruction with a comparison to healthy controls to determine if differences were present. Twenty-eight ACL injured participants and 28 matched healthy controls were included. They performed FL movements while sagittal plane biomechanics of the knee and electromyography (EMG) of nine leg muscles was assessed. The ACL injured group was tested before and 10 months after surgery. The perceived knee function and activity level was assessed by questionnaires. The ACL injured group performed the FL significantly slower than the controls before surgery (mean difference: 0.41 s [95%CI: 0.04–0.79 s; *p*<0.05]) while they performed the FL as fast as the controls after surgery (~28% movement time reduction post-surgery). Perceived knee function and activity level improved significantly post-surgery. The knee joint flexion angle, extensor moment, power, angular velocity in the ACL injured group did not differ from pre to post-surgery. For the ACL injured group, the peak knee extensor moment observed both pre and post-surgery was significantly lower when compared to the controls. The EMG results showed minimal differences. In conclusion, at 10 months post-surgery, the FL was performed significantly faster and the movement time was comparable to that of the controls. While the perceived knee function and activity level improved post-surgery, the knee joint biomechanics were unchanged. This may reflect that knee joint function was not fully restored.

## Introduction

Anterior cruciate ligament (ACL) rupture is a common injury that adversely affects the mechanical and sensory function of the knee joint. Surgical reconstruction of the ACL is a frequent treatment of ACL injured individuals where the goal is to reduce symptoms and restore normal knee stability and function [1–3]. While the post-operative patient-reported and functional outcomes of ACL reconstruction are generally good, the knee joint function is not fully restored [4, 5] and the rate of return-to-sport at the same level as pre-injury is modest [6, 7]. Ardern et al. 2011 reported that only 63% of the participants included in their systematic review managed to return to their pre-injury level of sports participation after ACL reconstruction.

Several cross-sectional studies have shown that the biomechanics and muscle activation patterns during various movements differ between healthy and ACL injured populations [8, 9]. However, the knowledge about differences in the movement pattern and muscle activation based on follow-up testing before and after ACL reconstruction is scarcer. In addition to patient-reported outcome measures (PROMS) and knee joint impairment assessments such as laxity and strength, a biomechanical movement pattern analysis may provide further insight into changes in the functional status after ACL reconstruction [10].

We have previously observed that ACL deficient subjects perform a forward lunge (FL) movement significantly different from healthy controls [11]. This study included copers (able to return to pre-injury sport activities without ACL reconstruction) and non-copers (unable to return to pre-injury sport activities) [11]. Both copers and non-copers were able to perform the FL without any pain or discomfort. The most conspicuous difference was that the non-copers performed the FL significantly slower (27%) than the healthy controls whereas the copers were as fast as the controls. Furthermore, the copers performed their FL movement with a biomechanical pattern close to that of the healthy controls, whereas the pattern of non-copers differed and was characterized by a significant reduction in the knee extensor moment [11].

During an FL movement, the posterior cruciate ligament is the primary passive stabilizer of the knee joint, while the mechanical role of the ACL seems to be modest [12–14]. Thus, in case of ACL deficiency, the FL movement is safe to perform, at least from a mechanical viewpoint. In addition, the FL movement has acceptable test-retest reliability [15]. Therefore, it seems appropriate to apply this dynamic movement in follow-up testing of the functional performance and biomechanics after ACL injury and ligament reconstruction.

Accordingly, the purpose of this study was to investigate the FL movement pattern before and after ACL reconstruction with a comparison to matched healthy controls to determine if differences were present.

## Materials and methods

### Participants

We included ACL injured participants clinically diagnosed and awaiting ACL reconstruction at Bispebjerg and Frederiksberg Hospital (Copenhagen, Denmark). Eligible participants were between 18 and 50 years of age, had a clinically verified ACL rupture, (positive Lachman test, positive pivot shift and increased anterior tibial translation measured with Rolimeter and compared to the healthy knee; confirmation during surgery), were free of pain in the lower extremities, had no neurological/cardiovascular diseases, and were not pregnant. Healthy matched volunteers were recruited among colleagues and relatives of employees at the University of Copenhagen. Prior to participation, all participants gave their written informed consent to participate in the experiments. The study was approved by the local ethics committee for the Capital Region of Denmark (De Videnskabsetiske Komiteer for Region Hovedstaden, H-3-2013-126) and the University of Ottawa Ethics Board (H06-14-27), and was performed in accordance with the Helsinki II declaration.

### Procedure

The present study is a part of a larger study where the participants underwent a protocol consisting of an isometric force matching protocol [16, 17], followed by one- and two-legged squats, forward lunges, hops, side-cuts, and countermovement jumps [18, 19]. The order of the tasks was always the same. The present study focus on the recordings of the forward lunges.

Upon arrival to the laboratory, the participants were asked to fill out questionnaires about their perceived knee function: The Lysholm score [20], International Knee Documentation Committee (IKDC) subjective form [21], the Knee Injury and Osteoarthritis Outcome Scale (KOOS) [22] and the Knee Numeric-Entity Evaluation Score (KNEES–ACL) [23]. The participants’ level of activity was assessed by the Tegner score [24]. The participants were instructed to perform three FL movements at a self-selected pace by taking one step forward, placing the foot on the force plate (OR 6-5-1, AMTI, USA), flexing the knee to 90° and subsequently push themselves backwards into the starting position, while having their hands on the back of their head, the upper body perpendicular to the ground, and the opposite foot maintaining contact with the ground. Verbal feedback was provided by the research team if the FL was deemed inadequate and the repetition was repeated.

Each participant was instrumented with bipolar surface electrodes for electromyography (EMG; 2DT2 Foam Dual Pregelled Electrode, Multi BioSensors Inc., USA) over the following muscles: Rectus femoris (RF), vastus lateralis (VL), vastus medialis (VM), biceps femoris (BF), semitendinosus (ST), gastrocnemius medialis (GM), gastrocnemius lateralis (GL), tensor fascia latae (TFL), adductor muscle group (ADDUC) and gluteus medius (GMED) after careful skin preparation. The EMG signals were transmitted wirelessly (MQair, Marq-Medical, 2012, Farum, Denmark) and recorded at 1000 Hz on a laptop using supporting software (Fireworks, Marq-Medical, 2012, Farum Denmark). Maximal voluntary isometric contractions (MVICs) of all muscle groups were performed in separate trials to assess the maximum voluntary EMG amplitude of each of the muscles. Hip extension, flexion, adduction, and abduction were performed while standing in neutral position and effort was exerted against a strap placed above the ankle. Participants stood and raised to their toes for plantarflexion while resisting upward motion using wall mounted bars. Knee extension and flexion were performed against manual resistance while participants sat with their hip and knee flexed to 90 and 30 degrees, respectively.

Anthropometrics were measured on the participants and then a cluster marker set [25] was attached to anatomical landmarks to collect kinematic data using a 10-camera motion capture system (6 MX and 4 T series, Vicon, Nexus, v1.8.5, Oxford Metrics, Oxford, UK) at a sampling frequency of 100 Hz. The ground reaction force (GRF) data were recorded simultaneously at a frequency of 1000 Hz.

### Data analysis

The FL movement time was defined as the period where the forward stepping foot was in contact with the ground and determined from the vertical GRF signal. The EMG signals were high-pass filtered with a 20Hz 2^nd^ order dual Butterworth filter, full-wave rectified, and low-pass filtered with a 10Hz 2^nd^ order dual low-pass Butterworth filter. The marker position and GRF data were filtered at 15 Hz using a 4^th^ order zero lag Butterworth filter [26]. Three-dimensional inverse dynamics were used to calculate the knee joint angles and moments of the sagittal plane. The knee joint power was calculated as the scalar product of the knee joint moment and the knee joint angular velocity.

### Data reduction and normalization

Data from three FL movements were time normalized and averaged for each participant. The body mass was used for normalization of the knee joint moment (Nm/kg), knee joint power (Watt/kg), and GRFs (N/kg). The EMG activity recorded during the FL movement was normalized to the maximum EMG amplitude obtained during the MVICs (% maxEMG). The following input parameters to the statistical analyses were then extracted: the movement time, the peak GRFs, peak knee joint moment, peak knee joint angle, the average knee joint moment and joint angle within the first and last 25% of the movement phase, the peak eccentric/concentric knee joint angular velocity, the peak eccentric/concentric knee joint power, the mean and peak EMG amplitudes of each muscle, the perceived knee function and the level of activity scores.

### Statistics

Paired t-tests were used to compare the variables pre and post-surgery, i.e. the ACL deficient (ACLd) versus the ACL reconstructed (ACLr) condition. Student’s t-tests were used for comparisons of the healthy controls (CON) and the ACL injured group and this was done pre (ACLd versus CON) and post-surgery (ACLr versus CON). Unless otherwise stated the results are reported as means ± SD. Mean group differences are reported with 95% confidence interval (CI). The level of significance was set to 5%.

## Results

### Participants

Forty-seven ACL injured patients who met the inclusion criteria, were invited and accepted to participate (20 females, 27 males). Nineteen of these patients were excluded due to: 1) not completing the FL movement test before surgery (n = 4) or after surgery (n = 7), 2) poor motion capture data quality (n = 4), 3) pregnancy at the time of testing (n = 2), 4) no ACL reconstruction (n = 1) and 5) moving (n = 1).

Twenty-eight healthy matched (11 females, 17 males) were recruited as controls. Participants’ characteristics are summarized in Table 1.

**Table 1:** Participant characteristics.

	*pre surgery*		*post-surgery*			
Characteristic	ACLd (n = 28)		ACLr (n = 28)		Control (n = 28)	
Sex (females/males)	11/17		11/17		11/17	
Time since injury (months)	19.7	(29.6)	−	−	−	−
Time since surgery (months)	−	−	10.7	(1.7)	−	−
BMI (kg/m^2^)	24.2	(2.7)	24.4	(2.9)	23.7	(3.0)
Age (years)	28.8	(9.0)	29.8	(9.1)	27.0	(6.9)
Height (m)	1.75	(0.1)	1.75	(0.1)	1.77	(0.1)
Mass (kg)	74.5	(10.9)	75.3	(11.6)	79.9	(11.9)

Values are mean (SD) for age, height, mass and sex are number of males and females.

At the pre surgery test time, the average [range] number of months since injury was 19.7 [1 to 120] months. The time from surgery to the post-surgery test was on average 10.7 [7 to 14.5] months (Table 1).

Most of the ACL injured participants underwent a double-bundle hamstring autograft reconstruction procedure (11 females, 13 males), whereas in two males a bone-patella-bone autograft was used, in one male an Achilles tendon allograft and one male an iliotibial band autograft was used.

### Movement time

**Fig 1:**
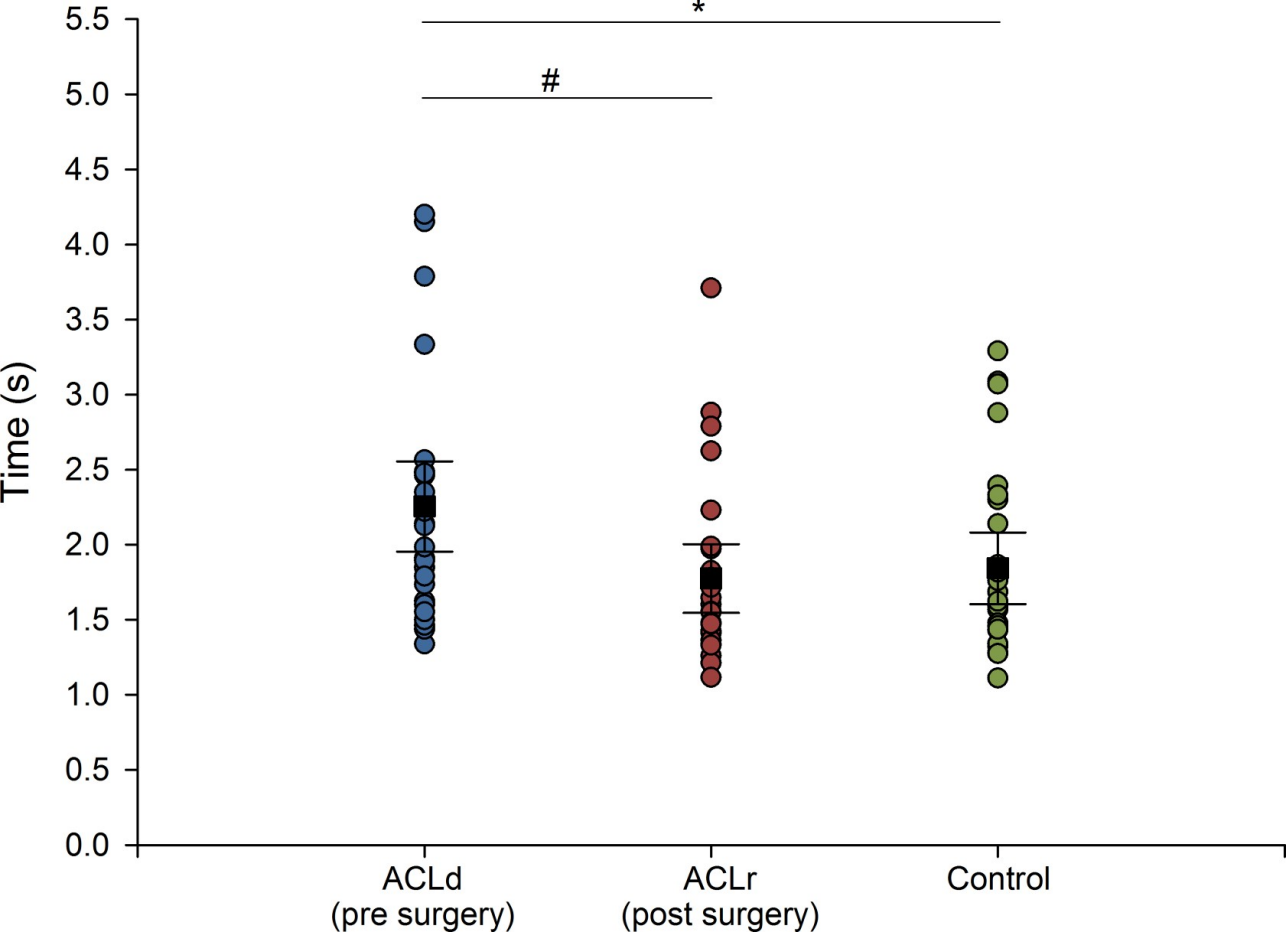
**Individual and group mean FL movement time (s) of controls (n = 28, green dots), ACLd (n = 28, pre surgery, blue dots) and ACLr (n = 28, post-surgery, red dots).** Group mean (black squares) with 95% CI. * indicates statistically significant difference between ACLd and controls. # indicates statistically significant difference between ACLd and ACLr (i.e. pre and post-surgery).

Prior to surgery, the ACLd group performed the FL movement significantly slower (~28%) than the controls (mean difference ACLd—CON: 0.41 s [95%CI: 0.04–0.79 s; *p*<0.05]; Fig 1). After surgery, the ACLr group performed the FL significantly faster than before surgery (mean difference ACLd—ACLr: 0.48 s [95% CI: 0.30–0.66 s; *p*<0.001]; Fig 1). The movement time observed after surgery did not differ significantly from that of the controls (mean difference ACLr—CON: -0.07 s [95%CI: -0.39–0.25 s, *p*>0.05]; Fig 1).

### Knee joint biomechanics and GRFs

The peak knee flexion angle was significantly reduced post-surgery (ACLr) when compared to the controls (Table 2). No other significant differences were noted. On average, the ACLr group reached a peak knee joint flexion angle of at least 90°, as they were asked to target during the experiments; however, the peak knee joint angle values of this group ranged from [-125.3° to -82.9°] and further investigation revealed that three ACLr participants had peak knee flexion values below 90°. In comparison, the range was [-120.1° to -85.6°] in the ACLd with two participants not reaching 90° while the range of the controls was [-126.2° to -92.1°] indicating that all the healthy participants flexed their knee more than 90°.

**Table 2:** Forward lunge biomechanics.

	Mean ± SD			Mean difference (95% CI)					
	ACLd (n = 28)	ACLr (n = 28)	Control (n = 28)	ACLd—ACLr ^a^	P value	ACLd—Control ^b^	P value	ACLr—Control ^b^	P value
**Peak GRFs (N/kg)**									
Vertical GRF (Fz)	8.6 ± 0.6	9.0 ± 1.3	9.3 ± 1.5	-0.4 (-0.9 to 0.2)	0.192	-0.7 (-1.3 to -0.05)	*0*.*034*	-0.3 (-1.1 to 0.4)	0.414
Horizontal ant-post GRF (Fy1)	-2.5 ± 0.5	-2.5 ± 0.4	-2.6 ± 0.6	0.1 (-0.3 to 0.1)	0.410	0.0 (-0.3 to 0.3)	0.898	0.1 (-0.2 to 0.4)	0.496
Horizontal ant-post GRF (Fy2)	-3.0 ± 0.8	-3.3 ± 0.8	-3.5 ± 0.9	0.2 (-0.1 to 0.5)	0.162	0.5 (0.0 to 0.9)	*0*.*049*	0.3 (-0.2 to 0.7)	0.264
**Knee joint angle (deg)**									
1–25%	-56.7 ± 9.2	-53.8 ± 7.9	-57.8 ± 10.1	-2.6 (-6.5 to 1.4)	0.188	1.0 (-4.2 to 6.3)	0.692	4.0 (-1.0 to 8.9)	0.056
75–100%	-64.2 ± 12.5	-60.7 ± 8.6	-64.8 ± 14.3	-3.5 (-7.4 to 0.5)	0.081	0.6 (-6.6 to 7.9)	0.861	4.1 (-2.3 to 10.5)	0.203
Peak flexion	-105.3 ± 9.5	-104.2 ± 9.7	-109.2 ± 7.9	-1.0 (-4.3 to 2.2)	0.509	4.0 (-0.8 to 8.7)	0.098	5.0 (0.2 to 9.7)	*0*.*041*
**Peak knee joint angular velocity (deg·s**^**-1**^**)**									
Eccentric phase	-314.6 ± 96.6	-332.6 ± 100.6	-419.7 ± 128.5	15.7 (-5.0 to 36.4)	0.131	105.2 (43.5 to 166.8)	*0*.*001*	87.1 (24.5 to 149.7)	*0*.*007*
Concentric phase	330.2 ± 87.8	358.2 ± 90.3	405.5 ± 115.0	-21.9 (-53.2 to 9.3)	0.160	-75.3 (-130.8 to -19.8)	*0*.*009*	-47.4 (-103.4 to 8.7)	0.096
**Knee joint moment (Nm/kg)**									
1–25%	0.3 ± 0.2	0.2 ± 0.2	0.4 ± 0.2	0.03 (-0.04 to 0.1)	0.333	-0.2 (-0.3 to -0.05)	*0*.*006*	-0.2 (-0.3 to -0.1)	*0*.*000*
75–100%	0.3 ± 0.3	0.2 ± 0.2	0.4 ± 0.3	0.06 (-0.03 to 0.1)	0.187	-0.1 (-0.3 to 0.04)	0.153	-0.2 (-0.3 to -0.03)	*0*.*018*
Peak moment	0.7 ± 0.2	0.6 ± 0.2	0.8 ± 0.2	0.06 (-0.02 to 0.1)	0.131	-0.1 (-0.3 to -0.02)	*0*.*020*	-0.2 (-0.3 to -0.1)	*0*.*000*
**Peak knee joint power (Watt/kg)**									
Eccentric phase	-1.7 ± 0.8	-1.7 ± 0.9	-2.6 ± 1.7	-0.0 (-0.4 to 0.4)	0.993	0.9 (0.2 to 1.7)	*0*.*011*	0.9 (0.2 to 1.7)	*0*.*011*
Concentric phase	1.9 ± 1.3	1.6 ± 1.2	2.7 ± 1.6	0.3 (-0.1 to 0.7)	0.185	-0.7 (-1.5 to 0.1)	0.068	-1.0 (-1.8 to -0.3)	*0*.*010*

^a^ Paired t-tests were applied to detect differences between variables between pre and post-surgery (ACLd—ACLr).

^b^ Unpaired t-tests were applied to detect differences between variables between controls and pre surgery (ACLd—Control) and post-surgery (ACLr—Control).

The knee joint moments of the control group were significantly higher than both the pre- and post-surgery ACL group, and no change occurred after surgery in the ACL group (Table 2). Likewise, the knee joint power and knee joint angular velocity observed both pre and post-surgery were significantly lower than those of the controls and with no statistically significant differences between pre and post-surgery (Table 2).

The peak GRF values of the ACL injured group were not statistically different between pre and post-surgery (Table 2). The peak GRF values observed before surgery (ACLd) were significantly lower when compared to the controls whereas no statistically differences in the GRF values were observed between post-surgery (ACLr) and controls (Table 2).

### Muscle activity

The mean GM EMG amplitude was significantly greater for the ACLd when compared to CON (Table 3). The peak and mean GMED EMG amplitudes were significantly greater for the ACLd and ACLr when compared to the CON (Table 3). Due to bad signal quality the EMG results contained several missing data–especially regarding the GMED muscle (see footnotes of Table 3). No other significant differences were observed.

**Table 3:** Forward lunge muscle activities (%maxemg).

	Mean ± SD			Mean difference (95% CI)					
	ACLd (n = 28)^c^	ACLr (n = 28)^c^	Control (n = 28)^c^	ACLd—ACLr ^a^	P value	ACLd—Control ^b^	P value	ACLr—Control ^b^	P value
**TFL**									
Mean amplitude (% maxemg)	9.7 ± 6.4	9.6 ± 6.0	7.8 ± 4.9	0.1 (-2.7 to 2.9)	0.921	1.9 (-1.3 to 5.2)	0.243	1.8 (-1.4 to 4.9)	0.260
Peak amplitude (% maxemg)	25.0 ± 16.1	24.1 ± 12.4	18.7 ± 10.6	0.9 (-4.9 to 6.6)	0.749	6.3 (-1.5 to 14.0)	0.110	5.4 (-1.3 to12.0)	0.110
**RF**									
Mean amplitude (% maxemg)	14.5 ± 9.3	13.3 ± 6.5	14.9 ± 11.0	1.2 (-2.8 to 5.1)	0.551	-0.4 (-6.2 to 5.3)	0.877	-1.6 (-6.8 to 3.6)	0.537
Peak amplitude (% maxemg)	32.5 ± 20.9	33.6 ± 15.1	35.5 ± 24.1	-1.0 (-11.4 to 9.4)	0.840	-2.9 (-15.7 to 9.9)	0.648	-1.9 (-13.4 to 9.6)	0.741
**VL**									
Mean amplitude (% maxemg)	25.6 ± 17.1	23.2 ± 11.3	24.3 ± 13.6	2.4 (-3.3 to 8.0)	0.395	1.3 (-7.4 to 10.0)	0.764	-1.1 (-8.1 to 6.0)	0.763
Peak amplitude (% maxemg)	62.2 ± 34.6	59.7 ± 24.0	54.4 ± 26.6	2.4 (-9.4 to 14.2)	0.676	7.7 (-9.6 to 25.0)	0.373	5.3 (-9.0 to 19.6)	0.458
**VM**									
Mean amplitude (% maxemg)	26.1 ± 17.3	21.4 ± 9.8	26.1 ± 12.7	4.7 (-1.4 to 10.8)	0.125	-0.1 (-8.6 to 8.5)	0.990	-4.7 (-11.2 to 1.8)	0.149
Peak amplitude (% maxemg)	62.1 ± 30.9	58.0 ± 24.9	59.0 ± 25.7	4.1 (-8.3 to 16.5)	0.500	3.1 (-13.1 to 19.2)	0.705	-1.0 (-15.3 to 13.3)	0.882
**BF**									
Mean amplitude (% maxemg)	6.7 ± 4.7	6.4 ± 3.4	5.6 ± 3.4	0.3 (-1.4 to 1.9)	0.731	1.1 (-1.2 to 3.4)	0.333	-0.8 (-1.1 to 2.8)	0.381
Peak amplitude (% maxemg)	19.1 ± 13.1	18.9 ± 11.5	14.9 ± 9.0	2.1 (-4.0 to 4.5)	0.920	4.2 (-2.1 to 10.5)	0.186	4.0 (-1.8 to 9.8)	0.174
**ST**									
Mean amplitude (% maxemg)	9.6 ± 8.3	10.1 ± 7.9	8.3 ± 6.4	-0.5 (-4.1 to 3.1)	0.783	1.3 (-2.9 to 5.5)	0.537	1.8 (-2.3 to 5.8)	0.382
Peak amplitude (% maxemg)	27.9 ± 22.6	25.5 ± 14.9	20.7 ± 13.1	2.4 (-6.5 to 11.3)	0.580	7.2 (3.1 to 17.5)	0.165	4.8 (-3.2 to 12.8)	0.231
**GL**									
Mean amplitude (% maxemg)	17.2 ± 9.8	13.6 ± 5.5	15.5 ± 8.2	3.6 (-0.7 to 7.8)	0.096	1.7 (-3.5 to 6.9)	0.514	-1.8 (-6.0 to 2.3)	0.379
Peak amplitude (% maxemg)	46.6 ± 21.4	41.0 ± 17.5	39.2 ± 21.7	5.6 (-6.5 to 17.7)	0.349	7.4 (-5.1 to 19.8)	0.240	1.8 (-9.7 to 13.3)	0.758
**GM**									
Mean amplitude (% maxemg)	15.4 ± 11.3	12.1 ± 7.4	9.6 ± 6.6	3.3 (-1.7 to 8.2)	0.183	5.4 (0.3 to 10.5)	*0*.*039*	2.1 (-1.8 to 6.0)	0.281
Peak amplitude (% maxemg)	52.5 ± 36.8	40.9 ± 23.7	36.8 ± 21.4	11.6 (-5.2 to 28.5)	0.167	15.7 (-0.9 to 32.4)	0.063	4.1 (-8.5 to 16.7)	0.516
**ADDUC**									
Mean amplitude (% maxemg)	11.3 ± 8.5	9.9 ± 8.6	11.7 ± 7.7	1.4 (-3.4 to 6.2)	0.551	-0.4 (-5.0 to 4.1)	0.852	-1.8 (-6.4 to 2.7)	0.422
Peak amplitude (% maxemg)	30.4 ± 18.4	25.1 ± 18.6	28.2 ± 20.4	5.4 (-5.2 to 15.9)	0.306	2.2 (-8.6 to 13.0)	0.684	-3.4 (-14.0 to 7.8)	0.565
**GMED**									
Mean amplitude (% maxemg)	18.9 ± 9.6	17.3 ± 7.1	11.3 ± 8.3	1.6 (-2.8 to 5.9)	0.460	7.6 (1.8 to 13.4)	*0*.*012*	6.1 (0.9 to 11.2)	*0*.*023*
Peak amplitude (% maxemg)	44.3 ± 22.2	37.5 ± 13.2	28.4 ± 17.9	7.2 (-2.5 to 16.9)	0.133	16.2 (3.2 to 29.3)	*0*.*016*	9.0 (-1.7 to 19.7)	0.095

^a^ Paired t-tests were applied to detect differences between variables between pre and post-surgery (ACLd—ACLr).

^b^ Unpaired t-tests were applied to detect differences between variables between controls and pre surgery (ACLd—Control) and post-surgery (ACLr—Control).

^c^ Missing EMG data: TFL: ACLd & ACLr (n = 6), CON (n = 1); RF: ACLd & ACLr (n = 4), CON (n = 1); VL: ACLd & ACLr (n = 3), CON (n = 2); VM: ACLd & ACLr (n = 4), CON (n = 2); BF & ST: ACLd & ACLr (n = 5), CON (n = 1); GL: ACLd & ACLr (n = 6), CON (n = 1); GM: ACLd & ACLr (n = 3), CON (n = 1); ADDUC: ACLd & ACLr (n = 3), CON (n = 1); GMED: ACLd & ACLr (n = 12), CON (n = 5).

### Perceived knee function and level of activity

Statistically significant differences were observed between ACLd and ACLr across all questionnaires indicating that the perceived knee function was improved after ACL reconstruction (Table 4). However, two of seven subscales of the KNEES-ACL (“symptoms” and “sport physical”) and three of five KOOS subscales (“symptoms”, “pain”, “activities of daily living”) did not significantly differ pre to post-surgery (Table 4).

**Table 4:** Patient reported outcome measures and level of activity pre (ACLd) and post-surgery (ACLr).

	Mean ± SD		Mean difference (95% CI)		
	ACLd (n = 28)	ACLr (n = 28)	ACLd—ACLr ^a^		P value
**Questionaire**					
Lysholm score ^b^	73.4 ± 11.2	84.3 ± 11.5	-11.0	(-16.5 to -5.5)	*<0*.*001*
IKDC ^b^	71.0 ± 10.0	81.9 ± 9.6	-10.7	(-14.5 to -6.9)	*<0*.*001*
KNEES-ACL activities of daily living ^c^	6.6 ± 4.5	3.8 ± 3.1	2.8	(1.2 to 4.4)	*0*.*001*
KNEES-ACL psychosocial ^c^	5.7 ± 3.9	3.4 ± 3.6	2.2	(0.8 to 3.6)	*0*.*004*
KNEES-ACL symptoms ^c^	4.8 ± 3.6	3.3 ± 3.5	1.4	(-0.2 to 2.3)	0.075
KNEES-ACL slackness ^c^	7.8 ± 3.3	5.0 ± 4.8	2.9	(0.8 to 5.1)	*0*.*010*
KNEES-ACL looseness ^c^	4.4 ± 2.4	2.4 ± 2.4	2.2	(0.8 to 3.5)	*0*.*003*
KNEES-ACL sports behaviour ^c^	13.1 ± 4.1	8.7 ± 5.3	4.0	(2.3 to 6.6)	*0*.*001*
KNEES-ACL sports physical ^c^	3.8 ± 3.8	2.9 ± 3.1	0.9	(-0.8 to 2.5)	0.308
KOOS symptoms ^b^	81.3 ± 12.2	82.1 ± 12.1	-0.4	(-6.9 to 6.1)	0.907
KOOS pain ^b^	82.4 ± 14.3	87.6 ± 10.8	-5.0	(-10.3 to 0.3)	0.064
KOOS activities of daily living ^b^	91.0 ± 9.7	91.8 ± 10.5	-1.0	(-4.1 to 2.1)	0.516
KOOS sports/recreation ^b^	57.6 ± 24.2	70.8 ± 24.9	-13.4	(-24.6 to -2.2)	*0*.*021*
KOOS quality of life ^b^	41.7 ± 12.1	58.9 ± 16.5	-18.0	(-26.0 to -10.0)	*<0*.*001*
**Level of activity**					
Tegner ^d^	4.2 ± 1.2	5.5± 1.4	-1.3	(-2.0 to -0.7)	*<0*.*001*

^a^ Paired t-tests were applied to detect differences between variables between pre and post-surgery (ACLd—ACLr).

^b^ Score range from 0–100 where higher scores represent higher levels of knee function.

^c^ The individual subscales ranges of KNEES-ACL are: activity of daily living 0–24; psychosocial 0–15; symptoms 0–18; slackness 0–21; looseness 0–12; sports behaviour 0–18; sports physical 0–12 where higher values represent lower levels of knee function.

^d^ Score range from 0–10 where higher levels represent higher activity level.

After ACL reconstruction, the level of activity increased by one Tegner score level and this difference was statistically significant (Table 4).

## Discussion

This exploratory study aimed to investigate the FL movement pattern before and after ACL reconstruction with a comparison to healthy controls to determine if differences were present. The main findings showed that the movement time and perceived knee function differed significantly from pre to post-surgery, while the peak knee joint extensor moment and power were unchanged in the ACL injured group. Before surgery, the ACL injured group performed the FL movement significantly slower than the controls, whereas after surgery they performed it as fast as the controls corresponding to a 28% reduction of the movement time. Furthermore, the knee joint extensor moment and power observed both pre and post-surgery were significantly lower when compared to the controls. In general, the EMG results showed minimal differences.

The faster FL movement performance after ACL reconstruction fit well with the observed improvements in perceived knee function and level of activity, but it was unaccompanied by changes in the knee joint dynamics. The knee joint extensor moment did not change significantly from pre to post-surgery and the comparison to the controls showed that this parameter was significantly lower for the ACL injured group both before and after surgery. This is well in line with our previous study [11] where ACL injured non-copers moved 27% slower and with a significantly reduced knee extensor moment during forward lunging compared to healthy controls. Reduced knee extensor moment during dynamic movements seems to be a general characteristic for ACL deficient non-coper subjects [27, 28]. The ACL injured participants in the present study can be equated with non-copers as their condition required surgical treatment. In addition, the non-copers of our previous study had mean Lysholm and Tegner scores of 74.0 (SD, 7.1) and 3.8 (SD, 0.6), respectively [11], which are comparable to the values reported before surgery by the ACL injured participants of the present study (see Table 4, ACLd column).

The observed mean change in Lysholm score from pre to post-surgery of 11.0 points (Table 4), is clinically relevant, as the minimally clinically important difference is 10.0 points in these patients [29]. Generally, the results presented in Table 4 reflect improved perceived knee function and level of activity across the different questionnaires. It is possible that the improved perceived knee function after surgery was facilitated by better control (mechanical stability) of the knee joint in the ACL injured group, and that this explains why the FL movement was performed faster after ACL reconstruction. However, the knee joint extensor moment and power did not change significantly after surgery and remained significantly reduced when compared to the healthy controls. ACL deficient copers have been observed to move as fast as healthy controls and with very similar knee joint dynamics [11], so a significant increase of the knee joint moment and power to drive the faster FL movement observed postoperatively would be expected. Therefore, other factors may explain the faster FL movement observed after surgery in the present study. Firstly, the ACL reconstructed patients possibly trusted and controlled their knee joint better because of the mechanical stability supplied by surgery, enabling faster FL movement performance. Secondly, during rehabilitation after surgery the patients may have developed a movement strategy that involves other joints (ankle and hip—perhaps also the contralateral leg) that benefits the movement performance. In support of this, the GRFs (vertical and horizontal 2^nd^ peak, Table 2) were significantly lower in the ACL injured participants before surgery when compared to the healthy controls, whereas this difference disappeared when comparing the post-surgery observations to the controls. However, there was no significant differences between GRF observations before and after surgery for the ACL injured group. Thus, it is impossible to conclude that the GRFs increased after surgery. Thirdly, the ACL injured participants flexed their knee joint less than the controls after surgery, which could reduce the movement time as well as the knee joint extensor moment. It is possible that the decreased knee flexion post-surgery may be due to increased quadriceps muscle strength deficit, which have been observed to persist even one year after ACL reconstruction [30]. As the ACL, injured participants in the present study were tested on average 10 months after ACL reconstruction it is possible their quadriceps muscle strength was not fully recovered. In support of this, a few PROMS subscales were not statistically significant post-surgery, e.g. KNEES-ACL “symptoms” and “sport physical” and most of the KOOS subscales (Table 4), which indicates that some functional deficits persisted in the ACL reconstructed group. In contrast, the EMG results did not reflect any quadriceps muscle activation deficit, as the activity level of these muscles appeared to be comparable between the ACL injured (pre and post-surgery) and the matched healthy controls. This presupposes that we were able to obtain the maximal EMG amplitudes in all muscles during MVICs as these were used for normalization of the EMG parameters. Finally, minimal differences were observed among the EMG results (Table 3) and those that were detected should be interpreted with caution because of many missing observations due to signals of poor quality (Table 3). However, the GMED muscle activity was significantly higher for the ACL injured participants when compared to the healthy controls and this was the case both before and after ACL reconstruction. It is possible that this is a compensatory mechanism to control the trunk and pelvis in the frontal plane [31] or the frontal plane knee joint motion [32, 33] when the knee joint sensory function and quadriceps muscle strength is impaired.

The present study is part of a larger study protocol including more demanding dynamic tasks [19] and the results of this forward lunge analysis corroborated with our observed improved subjective knee function but with modest differences in the EMG activity and biomechanics of hops and side-cuts [19]. However, a number of ACL injured participants were unable to complete the more challenging tasks in the study by Smale et al. 2019b, which therefore relates to a different group of high-functioning ACL injured participants when compared to the participants of the present study.

### Limitations

This study has limitations. Firstly, out of 47 ACL injured participants initially included only 28 ACL were tested both before and after surgery. This could impair the statistical power and increase the risk of selection bias. However, looking at the mean differences and 95% confidence intervals (Tables 2–4) the risk of type II errors does not seem to be high. Secondly, The EMG results suffered from missing data and must be interpreted with caution. Furthermore, the normalization of the EMG signals to the maximum amplitude obtained from MVICs assumes that no activation deficits were present in the study sample. Thus, if some ACL injured participant were unable to fully activate their muscles—and that probably applies to some of the participants [34], this may mask potential differences between groups and conditions. However, the applied normalization procedure has shown to be a reliable method in studies including ACL injured participants [35, 36]. Thirdly, all the ACL injured participants were treated at the same hospital but not by the same surgeon nor with the same reconstruction technique. After the ACL reconstruction, they were recommended to follow the same rehabilitation program but this was not monitored or supervised in our study. As such, the influence of these factors is unknown. Finally, we focused on the sagittal plane knee joint biomechanics. However, ACL insufficiency is not just resulting in anterior laxity but most importantly in rotational instability of the knee [37]. Thus, to further describe and understand the faster FL movement post-surgery and unchanged knee joint moment/power we suggest applying a more detailed FL movement analysis of the whole body (including the contralateral leg and trunk) in a future study. The clinical implications of improved FL movement performance and perceived knee function in combination with unchanged knee joint extensor moment after surgery cannot be determined from the present study.

### Conclusion

This study investigated the FL movement pattern before and after ACL reconstruction with a comparison to healthy controls to determine if differences were present. At 10 months after ACL reconstruction, the FL movement was performed significantly faster indicated by a ~28% decrease of the movement time corresponding to the level of the controls. While the perceived knee function and activity level improved after surgery, the knee joint biomechanics and muscle activation were unchanged. This may reflect that knee joint function had not fully recovered 10 months after ACL reconstruction.

## Supporting information

S1 Dataset (XLSX)
